# Reversibility of Membrane *N*-Glycome of HeLa Cells upon Treatment with Epigenetic Inhibitors

**DOI:** 10.1371/journal.pone.0054672

**Published:** 2013-01-15

**Authors:** Tomislav Horvat, Martina Deželjin, Irma Redžić, Darko Barišić, Maja Herak Bosnar, Gordan Lauc, Vlatka Zoldoš

**Affiliations:** 1 Faculty of Science, University of Zagreb, Zagreb, Croatia; 2 Ruđer Bošković Institute, Zagreb, Croatia; 3 Faculty of Pharmacy and Biochemistry, University of Zagreb, Zagreb, Croatia; 4 Glycobiology Laboratory, Genos Ltd, Zagreb, Croatia; 5 Edith Cowan University, Perth, Australia; INSERM UMR S_910, France

## Abstract

Glycans are essential regulators of protein function and are now in the focus of research in many physiological and pathophysiological processes. There are numerous modes of regulating their biosynthesis, including epigenetic mechanisms implicated in the expression of glyco-genes. Since *N*-glycans located at the cell membrane define intercellular communication as well as a cellular response to a given environment, we developed a method to preferentially analyze this fraction of glycans. The method is based on incorporation of living cells into polyacrylamide gels, partial denaturation of membrane proteins with 3 M urea and subsequent release of *N*-glycans with PNGase F followed by HPLC analysis. Using this newly developed method, we revealed multiple effects of epigenetic inhibitors Trichostatin A, sodium butyrate and zebularine on the composition of *N*-glycans in human cells. The induced changes were found to be reversible after inhibitor removal. Given that many epigenetic inhibitors are currently explored as a therapeutic strategy in treatment of cancer, wherein surface glycans play an important role, the presented work contributes to our understanding of their efficiency in altering the *N*-glycan profile of cancer cells in culture.

## Introduction

Glycans (oligosaccharide chains) play important roles in diverse cellular functions of all eukaryotic cells. By attaching to backbones of numerous membrane and soluble proteins they induce structural changes, thereby regulating and modifying protein function. Functional consequences of alternative glycosylation of a particular protein can be drastic, as exemplified by the case of immunoglobulin G. Here, the attachment of a single additional monosaccharide can lead to various outcomes, including conversion of IgG from pro-inflammatory into an anti-inflammatory agent [1] or activation of pathways implicated in various inflammatory diseases [2], [3]. Even though functional aspects of glycosylation of other proteins are less well understood, the importance of glycosylation in the regulation of biological activity of many other signaling and receptor proteins is certain. Amongst others, these are Notch, GLUT4 and NMDA receptor, whose glycosylation appears to play an important part in adaptive regulation of the cell surface in cell-cell adhesion and cellular communication [4].

Biosynthesis of glycan and polypeptide parts of a glycoprotein is different. Contrary to polypeptide moieties, glycan moieties of glycoproteins are not synthesized from the direct genetic template. Instead, glycan structures result from the activity of a dynamic network of over 600 glyco-genes [5] that code for various glycosyltransferases, glycosidases, enzymes for sugar nucleotide biosynthesis, transporters, etc. [6], [7]. Glycan biosynthesis can also be influenced by modulation of gene expression through epigenetic mechanisms as well as a change in the activity and/or the localization of any of the enzymes and various transcription factors, proton pumps, and other proteins involved in this complex pathway.

Epigenetic regulation of expression of glyco-genes is an obvious mechanism which can explain both the temporal stability of the glycome in healthy individuals [8] as well as specific changes which were reported to appear in various diseases [9], [10]. The number of studies on epigenetic factors involved in protein glycosylation is still limited [11], [12]. Nevertheless, ones that link epigenetics to glycosylation, mostly reporting aberrant glycosylation events in cancer [13 and ref herein], emphasize the importance of epigenetic factors in the regulation of protein glycosylation. For instance, promoter methylation was shown to regulate the expression of various glyco-genes, including α1,3-N-acetylgalactosaminyltransferase, an enzyme responsible for expression of the A determinant in the blood group A [14]; N-acetylglucosaminyltransferases GnT-IVa and GnT-IVb in pancreas [15]; α-1,3/4 fucosyltransferase (FUT) 3 in gastric carcinoma cell lines [16] and FUT7 in leukocytes [17]. Similarly, histone acetylation proved essential in the control of gene expression of α2,6 sialyltransferase (ST6GalNAc6), involved in the expression of sialyl Lewis^a^ antigen in cancers of the digestive organs, where it serves as a ligand for E-selectin, thus mediating metastasis [18].

The above-mentioned studies focus as well on the ability of various epigenetic inhibitors to restore the function of genes, silenced by aberrant epigenetic changes. It is this feature that makes them interesting candidates in epigenetic therapy. The most well known are the inhibitors of enzymes that establish and maintain DNA methylation patterns (DNA methyltransferases, DNMTs) and inhibitors of histone deacetylases (HDACs), which remove acetyl groups mostly from lysines of histones H3 and H4. Zebularine is a highly stable hydrophilic DNA methylation inhibitor [19], which preferentially depletes DNA methyltransferase 1 (DNMT1), as demonstrated in bladder, prostate, lung, colon, and pancreatic carcinoma cell lines [20], often resulting in inhibition of cell proliferation and induction of apoptosis [21], [22]. These effects are probably related to reactivated expression of epigenetically silenced genes both in carcinoma cells *in vitro* [21], [23], [24], as well as in tumors grown in mice [23], [25]. Zebularine exhibits low toxicity in mice even after prolonged administration [19], [26] and is the first in its class that can reactivate an epigenetically silenced gene by oral administration [23]. On the other hand, treatments of proliferating mammalian cells with deacetylase inhibitor Trichostatin A (TSA; [27]) induced various effects, including an increase in global H3K9 acetylation levels [28], relocation of pericentric heterochromatin towards the nuclear periphery [29], reactivation of growth-inhibiting genes [30] and altered expression of numerous glycan structures [31]. Even though 3 days long recovery from TSA treatment can result in complete restoration of histone acetylation levels, some induced changes were irreversible suggesting the existence of some type of epigenetic memory [32]. Another deacetylase inhibiting agent is sodium (Na)-butyrate, a non-toxic short-chain fatty acid. It has multiple effects on cultured mammalian cells including inhibition of proliferation, induction of differentiation and induction or repression of gene expression [33]. Interestingly, its strong proapoptotic action is reported in various types of cancer [34].

De-regulation of glycosylation was reported to occur in a wide range of diseases, including cancer, diabetes, cardiovascular, congenital, immunological and infectious disorders. By using limited glycoprofiling tools available to date, glycomic studies have revealed medically useful glycan biomarkers for cancer and other diseases [35]–[38]. Recently, we performed the first large-scale study of the human plasma glycome which revealed very high variability in the composition of plasma glycome in the population [39] and identified individuals having significantly aberrant glyco-phenotypes, some of which could be associated with specific diseases [40]. To test potential therapeutic usefulness of epigenetic inhibitors TSA, sodium butyrate and zebularine in inducing a reversal of undesired glyco-phenotypes, we developed an HPLC-based method for the determination of glycan structures from cells embedded in polyacrylamide gels. In addition, we specifically investigated the preservation of altered glycan profiles over a prolonged period of time in a drug-free environment. Our results emphasize the importance of epigenetic control in the regulation of *N*-glycosylation, but also suggest the stability of complex biosynthetic pathways responsible for the establishment of glycan profiles in human cells in culture.

## Materials and Methods

### Cell culture techniques and immobilization in polyacrylamide gel blocks

Human HeLa cell line (ATCC) was a kind donation of Dr. Marie-Lise Lacombe, Faculty of Medicine St. Antoine, Paris. Cells were cultured in Dulbecco's modified Eagle's medium (DMEM, Invitrogene) supplemented with 10% fetal bovine serum (FBS; Sigma Aldrich), 200 mM glutamine, 100 U/ml penicillin and 100 µg/mL streptomycin in humidified chamber with 5% CO_2_ at 37°C.

The cells (8×10^5^) were seeded in 35 mm cell culture plate and grown to confluence. Cells were detached with 1 mM EDTA, washed 3 times in phosphate buffered saline (PBS) and during the subsequent steps kept on ice. For cells lysate preparation the cells were treated with lysis buffer (25 mM Tris-HCl pH 7.5, 1% CHAPS, 5 mM EDTA), sonicated and dialyzed against 20 mM NH_4_HCO_3._ Vacum dried samples were mixed with reduction buffer (10 mM Tris-HCl pH 6.6, 40 mM DTT, 0.33% SDS) and incubated for 15 min at 65°C in order to reduce disulfide bonds. After reduction samples were alkylated by addition of 2 µL of 100 mM iodoacetamide (Sigma) and incubated for 30 min at RT in dark. Prepared samples were embedded in 70.5 µl of polyacrylamide gel (19.15% acrylamide:bisacrylamide 37.5:1, 478 mM Tris pH 8.8, 0.3% SDS, 0.3% APS, 1.5% TEMED). For the immobilization of intact cells, the cells were resuspended in 70 µL of polyacrylamide gel (18.5% acrylamide:bisacrylamide 29.2:0.2, 375 mM Tris-HCl pH 7.5, 3 M urea, 0.1% APS, 1.5% TEMED) and left to polymerize on ice between 1 and 2 minutes.

### Immunocytochemistry and confocal scanning microscopy of embedded cells

For confocal scanning microscopy the cells were embedded in polyacrylamide and fixed in 4% formaldehyde, washed 3–4 times in TBS (*50 mM Tris, 150 mM NaCl, pH 7.4*) and incubated with Biotinylated Ricinus Communis Agglutinin I (5 µg/ml; Vector Labs) in TBS over night, at 4°C. After incubation, gel was washed three times in TBS and incubated 1 h with Streptavidin-PE (0,125 µg/ml, BD Biosciences) in dark at RT. Gel was washed three times with TBS, mounted on a slide and covered with a coverslip. Fluorescent images were obtained using Leica TCS SP2 AOBS laser scanning confocal microscope equipped with HCXPL APO λ-Blue 63×1.4 objective.

### Glycan release and labeling

Gels were transferred into wells of UNIFILTER protein precipitation (PP) fast flow (FF) plate (Whatman, 96 well plate, 2 ml, glass polypropylene). 1 ml of acetonitrile was added to the wells and after 10 min of shaking, the liquid was vacuumed to waste. The washing procedure has been continued with 20 mM NaHCO_3_, ACN, 20 mM NaHCO_3_, and finished with ACN. UNIFILTER PP FF plate was then placed on a clean collection 96 well plate and gels were soaked with 1 µl of PNGase F (ProZyme *N*-glycanase; Peptide-*N*-glycosidase F 2.5 U/ml) diluted in 99 µl of 20 mM NaHCO_3_. Gels were covered with another 50 µl of 20 mM NaHCO_3_, sealed with adhesive sealing film and left to incubate for 18 h at 37°C. Samples which were left untreated with PNGase F were incubated with 100 μl of 20 mM NaHCO3 and further analysis has been performed as for PNGase F treated samples.

Released *N*-glycans were eluted from gels by washing with 200 µl water, shaking for 10 min, and collecting the liquid to the collection plate. The procedure was repeated two more times, and continued with 200 µl of ACN, 200 µl of water, and finished with 200 µl of ACN. Released *N*-glycans were then dried in vacuum centrifuge and fluorescently labeled with 2-aminobenzamide as described by Royle *et al*. [41]. Labeled glycans were dried in vacuum centrifuge and re-dissolved in known volume of water for further analysis.

### Hydrophilic interaction high performance liquid chromatography (HILIC)

Released glycans were subjected to hydrophilic interaction high performance liquid chromatography (HILIC) on a 250×4.6 mm i.d. 5 μm particle packed TSKgel Amide 80 column (Tosoh Bioscience, Stuttgart, Germany) at 30°C with 50 mM formic acid adjusted to pH 4.4 with ammonia solution as solvent A and acetonitrile as solvent B. 60 min runs were performed with fluorescence detector set with excitation and emission wavelengths of 330 and 420 nm, respectively. The system was calibrated using an external standard of hydrolyzed and 2-AB-labeled glucose oligomers from which the retention times for the individual glycans were converted to glucose units (GU).

### Exoglycosidase sequencing of glycans

The following enzymes, all purchased from ProZyme (CA, USA), were used for digestions: Sialidase A™/NANase III (recombinant gene from *Arthrobacter ureafaciens*, expressed in *E. coli*), 2.5 mU; α(1–2,3,4,6)fucosidase (bovine kidney), 1.16 mU; α(1–3,4)-fucosidase (almond meal), 3.2 μU; β(1–3,4)-galactosidase (bovine testis), 5 mU; β-N acetylhexosaminidase/HEXase I (recombinant gene from *Streptococcus pneumoniae*, expressed in *E. coli*), 40mU; α(1–2,3,6)-mannosidase (jack bean), 300 mU. Aliquots of the 2-AB labeled glycan pool were dried down and digested in a mixture of enzymes, corresponding 1X concentrated manufacturers buffer and water in total volume of 5 µl. After overnight incubation at 37°C, enzymes were removed by filtration through the AcroPrep 96 Filter Plates, 10K (Pall Corporation, MI, USA). Digested glycans were then separated by HILIC-HPLC for comparison against an undigested equivalent.

For HPLC analysis of mannosidase digested and non digested PNGase F free sample, we included an internal standard in order to equalize and normalize peak signals, correctly interpret background signals and verify the composition and percentages of oligomannose structures within these peaks. 2-aminobenzamide labeled dextran peak (120 000 glucose units), was added to both samples in equal amounts before enzyme removal by filtration trough AcroPrep 96 Filter Plates, 10K (Pall Corporation, MI, USA) and HPLC analysis. In this chromatography period we did not previously detect chromatography peaks in PNGase F free samples. Chromatography peaks were separated in same conditons for both samples.

### Treatment with Trichostatin A

Trichostatin A (Sigma) was dissolved in dimethyl sulfoxide at stock concentration of 1 mg/mL and stored at −20°C until use. HeLa cells were plated at a density of 6.4×10^5^ cells in 6 cm dishes 24 h prior to the treatment with 40 ng/mL TSA. 24 h after the treatment, the medium was replaced with fresh medium containing same concentrations of TSA. Cells were either collected and embedded in polyacrylamide gels 48 h post-treatment or, before embedding, left to recover in a fresh (drug-free) medium for 24 h or 5 days.

### Treatment with sodium (Na-) butyrate

Na-butyrate (Sigma) was dissolved in Phosphate buffered saline (PBS) at stock concentration of 0.5 M and stored at −20°C until use. HeLa cells were plated at a density of 6.4×10^5^ cells in 6 cm dishes 24 h prior to the treatment with 6 mM Na-butyrate. Cells were either collected and embedded in polyacrylamide gels 24 h post-treatment or, before embedding, left to recover in a fresh (drug-free) medium for additional 24 h.

### Treatment with zebularine

Zebularine (Sigma) was dissolved in Phosphate buffered saline (PBS) at a stock concentration of 10 mM and stored at 4°C until use. HeLa cells were plated at a density of 1.8 or 3.6×10^5^ cells in 6 cm dishes 24 h prior to the treatment with 100 μM zebularine. 48 h after the first treatment, the medium was replaced with fresh medium containing same concentrations of zebularine. Cells were either collected and embedded in polyacrylamide gels 72 h post-treatment or, before embedding, left to recover in a fresh (drug-free) medium for 72 h. Control cells were left untreated.

### Data analysis

The surface under each individual chromatographic peak, representing the contribution of a specific glycan group (GP1–GP12) in total measured glycans, was expressed as percentages. Changes in glycan contribution following treatments with diverse epigenetic inhibitors were normalized to control values, which were set to 100%.

## Results and Discussion

### 1. Quantification of glycans from embedded cells improves reproducibility of the analysis

**Figure 1:**
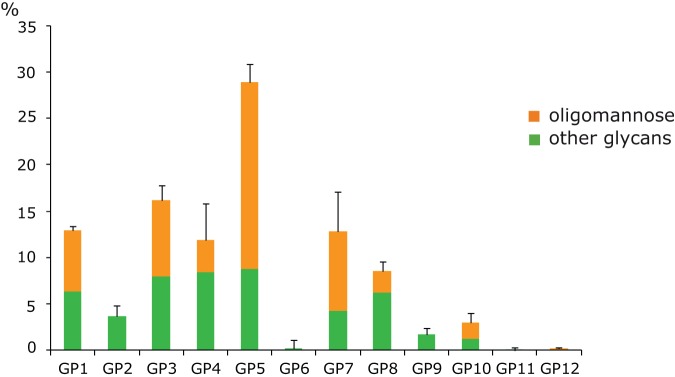
Contributions of individual glycan groups (GP1-GP12) to the cell membrane *N*-glycome. Contributions of mannose-bearing glycans within each individual group are colored in orange.

Following co-polymerization of HeLa cells in the acrylamide gels, *N*-glycans were released from the cell surface and labeled with 2-AB. The composition of the membrane glycome was expressed as the contribution of glycan groups eluted as individual chromatographic peaks (Fig. 1). In addition to glycan identification based on GlycoBase (http://glycobase.nibrt.ie) assignment, we performed exoglycosidase sequencing of the major HPLC peaks to confirm attributed structures (Fig. S1). Since our previous results suggested high content of oligomannose structures in the membrane glycome [31], we treated the sample with the enzyme mannosidase and confirmed significant contribution of oligomannose glycans to most of the glycan groups (Fig. 1). The effect was especially remarkable in case of GP5 (biantennary disialylated glycans with fucosylated core) and GP7 (tetraantennary glycans) where their relative contributions to the total *N*-glycome were reduced from 28,92% to 8,74%, and 12,83% to 4,23%, respectively. Since oligomannose glycans are involved in protein folding [42], their role in cancerous cells could easily be associated with improper maturation of membrane *N*-glycoproteins, thereby affecting cellular interactions with the environment. Indeed, it has been shown that the overexpression of glycoproteins with oligomannose *N*-glycans at the cell surface induces proliferative and adhesive properties of cancer cells and potentially facilitates loss of contact inhibition, a hallmark feature of majority of cancer cells [43].

**Figure 2:**
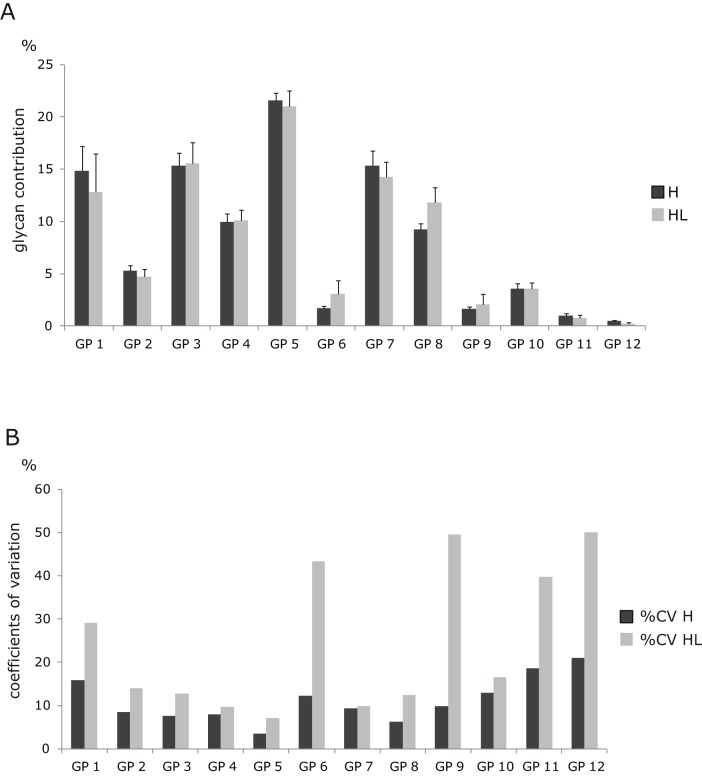
Composition of membrane and cell lysate *N*-glycomes. **A.** Glycans released from either HeLa cells embedded in polyacrylamide gels (H) or HeLa cell lysates (HL) were labeled with 2-AB and separated by HPLC. The histogram shows the partition (percentage + SD) of individual glycan fractions (GP1 – GP12) of HPLC separated glycome. **B.** Coefficients of variation (%CV) of individual glycan peaks in HPLC separated glycome of HeLa cells embedded in polyacrylamide gels (H) and HeLa cell lysates (HL) from 6 identical experiments.

Since glycans can also be obtained following disruption of cellular integrity, we decided to compare the methodological precision of *N*-glycan analysis from embedded cells to the total *N*-glycome obtained by the analysis of HeLa cell lysates. The observed glycan profiles were somewhat different, with particular glycan groups being selectively more abundant in the lysates or the embedded cell fraction (Fig. 2A). This is not surprising since Golgi and ER, which get disrupted during cell lysis, contain high amounts of both complete and partly synthesized glycans [44] and these can alter the proportion of a particular glycan group.

Each of the two glycan release methods was repeated six times to estimate their reproducibility. For nearly all glycan peaks, we observed higher coefficients of variation when glycome was analyzed from cell lysates (Fig. 2B). Especially high experimental variation was observed in highly branched glycan structures (GP9-GP12), which are important for the regulation of membrane half-life of many receptors [45], [46]. This increased variation of complex structures from experiment to experiment probably reflects their small contribution to the total glycome. Since glycans function as regulators of the activity of membrane proteins, many of them are also attached to proteins on the luminal side of various cytoplasmic vesicles, including the Golgi apparatus where posttranslational glycan processing occurs. Homogenization of cells results in mixing of these two compartments, containing physiologically separate fractions of glycans, and consequently leads to masking of relatively subtle, but functionally important, differences in the cell membrane *N*-glycome [45]. Therefore, we believe that the analysis of *N*-glycome from embedded cells results in improved analytical precision due to elimination of the homogenization step from the procedure. Based on these observations we decided to further exploit the method of glycan analysis from embedded cells.

### 2. Glycans extracted from embedded cells preferentially constitute a cell membrane fraction

**Figure 3:**
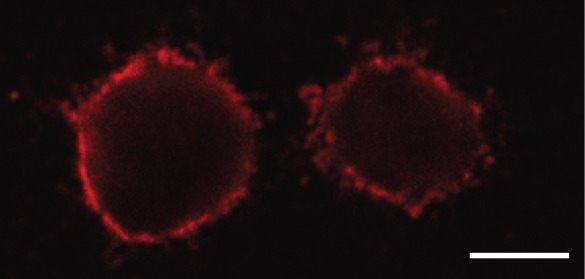
Enzymatically untreated HeLa cells embedded in polyacrylamide gels release glycan structures. Confocal images of HeLa cells embedded in polyacrylamide gel. The peripheral cellular glycans are stained with Ricinus Communis Agglutinin I. Scale bar is 5 µm.

**Figure 4:**
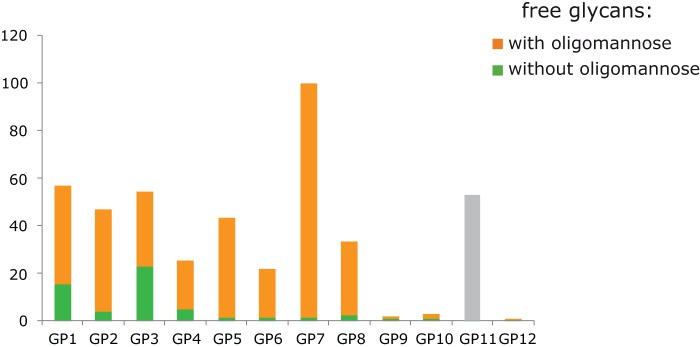
Mannosidase treatment of glycans released from enzymatically untreated HeLa cells. Column height of each individual glycan group is normalized to the highest one, set to 100 (GP7). Gray column (GP11) represents an internal standard (2-aminobenzamide labeled dextran peak) added in order to equalize and normalize peak signals, correctly interpret background signals and verify the composition and percentages of oligomannose structures within these peaks.

To define more accurately the origin of glycan fraction isolated from the embedded cells, we observed cells following their embedding into the acrylamide gels by confocal scanning microscopy. Staining with Ricinus Communis Agglutinin I confirmed the preserved integrity of the cell membrane during the embedding process (Fig. 3), thus arguing in favor of glycans originating mostly from the cell membrane glycoproteins. However, since we could not exclude the possibility of leakage during the subsequent steps, we analyzed glycans from embedded cells that were not treated with PNGase F, in order to release glycans from glycoproteins. Interestingly, we obtained certain glycan peaks and attributed those to oligomannose structures, due to an existing efflux of oligomannose glycans [47]. We validated this hypothesis by mannosidase treatment, which resulted in almost complete disappearance of the corresponding chromatographic peaks (Fig. 4), thus confirming the contribution of free oligomannose glycans to the pool of glycans released by PNGase F from glycoproteins associated with the cell membrane. In addition, a tiny fraction of remaining peaks (GP1 and GP3) could as well predominantly represent glycan structures transported to the cell surface unattached to their protein counterparts. Based on the presented experiments, we conclude that there was no significant glycan leakage from embedded cells, since the chromatogram obtained from the analysis of untreated cells did not contain structures other than the oligomannose.

### 3. Modulation of the HeLa cell membrane *N*-glycome by deacetylase inhibitors Trichostatin A and Na-butyrate

Histone acetylation at gene promoters is an important epigenetic modification controlling gene expression. Treatment with the deacetylase inhibitor Trichostatin A resulted in altered expression of glyco-genes in HepG2 cancer cells [48], whereas both Trichostatin A and sodium butyrate altered the composition of glycan structures in HeLa cells [31]. However, whether the original glycan profile upon drug removal from the medium is preferentially restored or irreversibly changed is still unknown.

**Figure 5:**
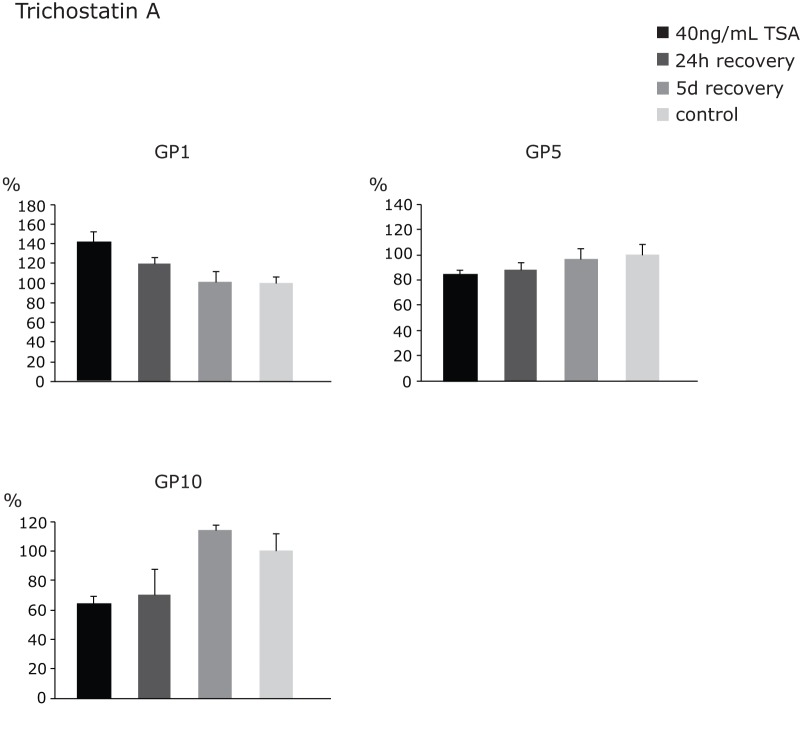
Changes in mean contributions of glycan groups GP1, GP5 and GP10 following TSA treatment and recovery. The observed changes were normalized to control values, which were set to 100%. The error bars represent standard deviations obtained on the basis of 4 independent experiments.

**Figure 6:**
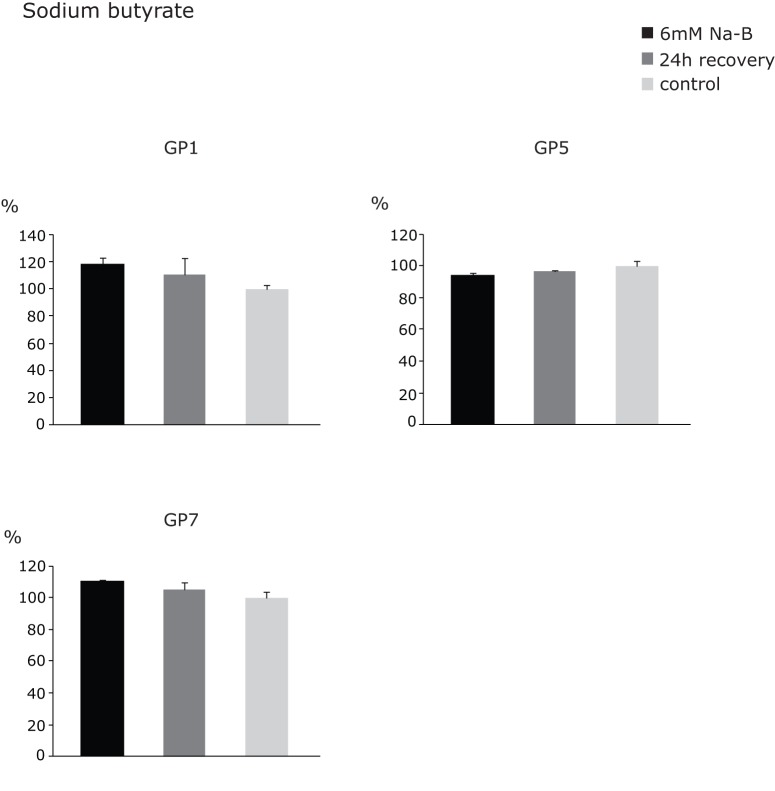
Changes in mean contributions of glycan groups GP1, GP5 and GP7 following Na-butyrate treatment and recovery. The observed changes were normalized to control values, which were set to 100%. The error bars represent standard deviations obtained on the basis of 3 independent experiments.

We performed multiple independent experiments wherein, following the treatments, HeLa cells were left to grow in a drug-free medium. Major N-glycome changes were not expected since the inhibition of HDAC activity affects the expression of only 2% of mammalian genes [49], [50]. However, a couple of glycan groups were affected (Fig. S2). In general, sodium butyrate induced milder changes than TSA (up to 20% versus up to 40%), probably due to its lower affinity to bind the substrate [51], requiring higher concentrations to have an effect [52]. Both inhibitors induced the increased expression of biantennary glycans (GP1), and the decreased expression of disialylated glycans with core-fucose (GP5) (Fig. 5 & 6). Specifically, TSA downregulated the expression of complex, branched glycan structures from GP10 (Fig. 5), whereas sodium butyrate upregulated the expression of tetraantennary glycans from GP7 (Fig. 6). Since incorporation of fucose into glycoproteins and increased glycan branching are both associated with malignant phenotype [53], [54], the observed reduction in quantity of these glycans following an epigenetic treatment is a desirable effect. However, following cell recovery in a drug-free medium we observed reversal of the induced effects and obtained similar composition of the membrane *N*-glycome of HeLa cells as prior to the treatments (Fig. 5 & 6). This finding strongly argues in favor of stability of the *N*-glycome and corresponding epigenetic mechanisms responsible for establishment and maintenance of histone acetylation levels of glyco-genes involved in glycan biosynthesis. It also confirms transient mode of TSA action, based on reversible binding to HDACs [55], [56], and demonstrates the importance of recurrent epigenetic treatments for successful reversal of potentially undesirable glycan phenotypes.

### 4. Modulation of the HeLa cell membrane *N*-glycome by zebularine

**Figure 7:**
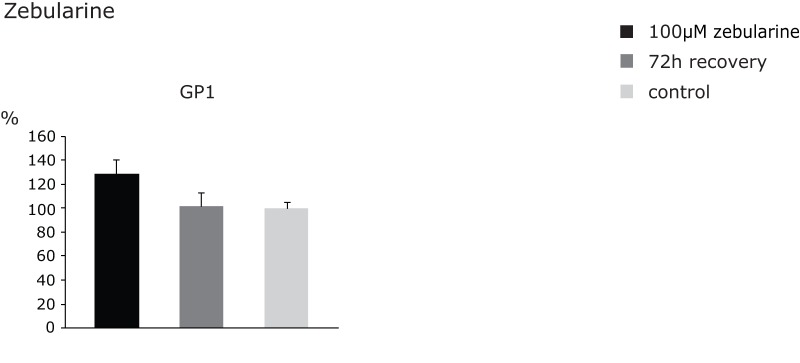
Changes in mean contribution of the glycan group GP1 following zebularine treatment and recovery. The observed changes were normalized to control values, which were set to 100%. The error bars represent standard deviations obtained on the basis of 4 independent experiments.

The role of DNA methylation in the regulation of glycosylation has particularly been studied in the context of fucosylation, which is present mostly on secretory or membrane proteins on the cell surface, such as epidermal growth factor and transforming growth factor beta receptors [57], [58]. Fucosylation levels in normal liver and colon are relatively low, but increase during carcinogenesis, mediating killing of oncogenically transformed cells by natural killer (NK) cells [59]. However, mutations in the gene GMDS (GDP-mannose-4,6-dehydratase) coding for a key enzyme involved in the synthesis of GDP-fucose, a donor substrate for fucosyltransferases, further enhance tumor growth due to developed resistance to NK cells [60]. Therefore, zebularine treatments of various cancer cell lines with relatively low fucosylation levels were performed in order to induce up-regulation of fucosylation-related genes and consequently restore global fucosylation level [57]. Interestingly, we observed in HeLa cells that the expression of fucosylated glycans (from GP2-GP6) was not altered following the 100 μM zebularine treatment (Fig. S2). This finding could imply either the existence of an alternative fucosylation-independent immune-protective mechanism related to cervical carcinoma or that the fucosylation level itself in HeLa cells is not significantly lowered as a result of malignant transformation, thus abrogating the need for its restoration to normal levels. Further insight into the matter could potentially be gained by investigating mutations (e.g. in GMDS) and expression levels of fucosylation-related genes followed by comparison of obtained results between cancerous and healthy tissue on one hand and cancer cells in culture on the other. In contrast to mostly unaffected fucosylated glycans, the simplest biantennary (GP1) glycans were strongly up-regulated following zebularine treatment (Fig. 7). However, the 72h-recovery in a drug-free medium resulted in almost complete restoration of normal values, arguing in favor of reversible covalent association between the DNMTs and the zebularine-containing DNA [61].

### 5. Conclusions

Here, we demonstrate the effects of three epigenetic inhibitors on the composition of *N*-glycome present preferentially at the surface of HeLa cells. Interestingly, all of the induced changes were reversible upon recovery in a drug-free medium. These observations argue in favor of transient stability of chemical bonds between the inhibitors and corresponding enzymes whose activity they abrogate. Since glycans play various important roles during carcinogenesis, we believe it is necessary to monitor changes in their expression levels as a result of treatments with epigenetic inhibitors, of which many are currently under clinical evaluation as promising antitumor therapeutic agents. Especially important fraction affecting cellular communication, whereby defining response to a diseased state/cell, is represented by glycans found at the cellular surface. Therefore, we argue that thorough understanding of the response of this glycan fraction to a given epigenetic treatment is at the basis of a successful medical therapy, facilitating choice of an adequate drug as well as appropriate dosage, duration of the treatment and frequency of drug administration.

## Supporting Information

Figure S1 **HPLC analysis of the HeLa cell membrane** ***N*** **-glycome.** Only the most abundant glycans in each HPLC glycan peak (GP) deduced by exoglycosidase digestions are shown. Black square presents N-acetylglucosamine, grey circle mannose, white circle galactose, triangle fucose and diamond presents sialic acid.
(EPS)

Figure S2 **Changes in HeLa cell membrane** ***N*** **-glycome induced by Trichostatin A (A), Na- butyrate (B) and zebularine (C), and N-glycome profile after recovery in a drug-free environment.** The error bars represent standard deviations.
(EPS)
